# 9,000 years of genetic continuity in southernmost Africa demonstrated at Oakhurst rockshelter

**DOI:** 10.1038/s41559-024-02532-3

**Published:** 2024-09-19

**Authors:** Joscha Gretzinger, Victoria E. Gibbon, Sandra E. Penske, Judith C. Sealy, Adam B. Rohrlach, Domingo C. Salazar-García, Johannes Krause, Stephan Schiffels

**Affiliations:** 1 Max Planck Institute for Evolutionary Anthropology, Department of Archaeogenetics, Leipzig, Germany; 2 Division of Clinical Anatomy and Biological Anthropology, Department of Human Biology, University of Cape Town, Cape Town, South Africa; 3 Department of Archaeology, University of Cape Town, Cape Town, South Africa; 4 School of Computer and Mathematical Sciences, University of Adelaide, Adelaide, South Australia Australia; 5 Department of Geological Sciences, University of Cape Town, Cape Town, South Africa; 6 Departament de Prehistòria, Arqueologia i Història Antiga, Universitat de València, València, Spain

**Keywords:** Archaeology, Population genetics, Population dynamics

## Abstract

Southern Africa has one of the longest records of fossil hominins and harbours the largest human genetic diversity in the world. Yet, despite its relevance for human origins and spread around the globe, the formation and processes of its gene pool in the past are still largely unknown. Here, we present a time transect of genome-wide sequences from nine individuals recovered from a single site in South Africa, Oakhurst Rockshelter. Spanning the whole Holocene, the ancient DNA of these individuals allows us to reconstruct the demographic trajectories of the indigenous San population and their ancestors during the last 10,000 years. We show that, in contrast to most regions around the world, the population history of southernmost Africa was not characterized by several waves of migration, replacement and admixture but by long-lasting genetic continuity from the early Holocene to the end of the Later Stone Age. Although the advent of pastoralism and farming substantially transformed the gene pool in most parts of southern Africa after 1,300 bp, we demonstrate using allele-frequency and identity-by-descent segment-based methods that the ‡Khomani San and Karretjiemense from South Africa still show direct signs of relatedness to the Oakhurst hunter-gatherers, a pattern obscured by recent, extensive non-Southern African admixture. Yet, some southern San in South Africa still preserve this ancient, Pleistocene-derived genetic signature, extending the period of genetic continuity until today.

## Abstract (web summary)

Oakhurst rockshelter in South Africa documents marked cultural change during the Holocene, but genome-wide data from ancient human individuals at the site now demonstrate a remarkable degree of genetic continuity over the last 9,000 years: the contemporary ‡Khomani San and Karretjiemense from South Africa still show direct signs of relatedness to the Oakhurst hunter-gatherers.

## Main

Southern African populations today harbour genetic variation that traces deep human population history^1,2^, reflected also in the archaeological record with fossils of archaic *Homo sapiens* dating back to 260 thousand years ago (ka) before present (bp) (uncalibrated) and evidence for the presence of anatomically modern humans in South Africa from at least ~120 ka bp onwards^3,4^. While genetic investigations have extensively explored the significance of southern African population structure in human evolution, there is a noticeable gap in our understanding of the more recent demographic trajectories during the Holocene (the last 11,700 years), which remain relatively understudied genetically.

During the Holocene, major transformations in lithic industries and subsistence practices probably also reflect demographic shifts^5,6^. In the last 2,000 years, the spread of pastoralism and farming have resulted in repeated admixture events visible in genetic complexity in both ancient and contemporary populations^1,2,7,8^. First, the spread of herders contributed northeast African, Levantine-enriched ancestry to the genetic make-up of southern African hunter-gatherers^2^. Second, the influx of farmers closely related to present-day Bantu-language speakers introduced western African ancestry to San and Khoe populations^2^. Consequently, all contemporary San and Khoe groups exhibit at least 9% genetic admixture from non-San sources outside modern-day South Africa, Namibia and Botswana^1,2,7,8^, obscuring the population structures of the Later Stone Age (LSA) San population. To provide insights into early Holocene San population structure, we sampled and recovered genome-wide data from a series of individuals unearthed from the Oakhurst rockshelter in South Africa, offering a chronological spectrum spanning most of the Holocene.

**Fig. 1:**
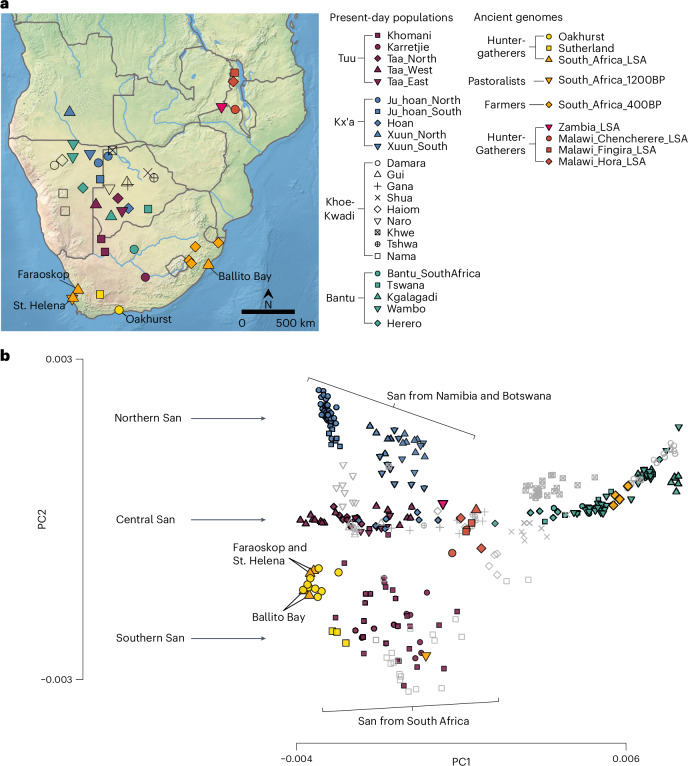
Population structure in present-day southern Africa. **a**, Approximate locations of present-day populations and ancient individuals mentioned in the article. Present-day populations are coloured according to linguistic affiliation, as indicated in the legend. **b**, PCA on 212,000 SNPs with ancient individuals projected onto PC1 and PC2. Shown are the positions of each individual along the first and second axes of genetic variation, with symbols denoting the individual's population and linguistic affiliation using the same colour coding as in **a**.

Oakhurst rockshelter is located close to George in southernmost Africa, ~7 km from the coast (Fig. 1a). Excavated in the first half of the twentieth century^9^, it is especially noteworthy for its substantial accumulation of deposit that spans 12,000 years. Its Early Holocene macrolithic stone artefact assemblage is characteristic of the period, similar to those found at many sites across South Africa and is today regarded as a distinctive technocomplex termed the ‘Oakhurst Complex’, named after the site^5,6,10–13^. At ~8,000 bp, the lithics change to microlithic ‘Wilton’ assemblages which persist through the Middle and Late Holocene, albeit with some temporal shifts, notably the addition of ceramics in the last 2,000 years (refs. ^6,9,14,15^). The site also preserves the complete and partial burials of 46 juvenile and adult individuals, spanning the complete period of occupation of the site^16,17^ and providing a valuable resource for the study of LSA San population structure. Here, we present genome-wide data from 13 individuals, including the oldest DNA from South Africa, dating back to ~10,000 years (calibrated) cal bp.

## Results

We sampled skeletal remains from 13 individuals, each radiocarbon dated on bone collagen with dates ranging between approximately 10,000 and 1,300 cal bp (Supplementary Table 1). Nine of these ^14^C dates were previously reported; for four samples we generated new ^14^C dates. We prepared powder from skeletal material, extracted ancient DNA (aDNA) and converted it into double- or single-stranded libraries (Methods; Supplementary Table 1). We selected 11 double-stranded and 15 single-stranded libraries for hybridization DNA capture to enrich for sequences that overlapped 1.24 million single-nucleotide polymorphisms (SNPs). For all 13 individuals, we determined the genetic sex and classified mitochondrial DNA and Y chromosome haplogroups for nine and five individuals, respectively, all of which are common in ancient and contemporary San and Khoe populations^1,2,18–24^ (Supplementary Table 1). After quality filtering (Methods) and merging of duplicate libraries, we recovered genome-wide data sufficient for population genetic analysis for nine individuals, featuring on average 368,359 SNPs of the 1,240k panel, a mean mtDNA contamination of 1.7% and a mean X chromosome contamination of 1.5% (Supplementary Table 1).

### Genetic affinities between Oakhurst and contemporary San and Khoe

**Extended Data Fig. 1:**
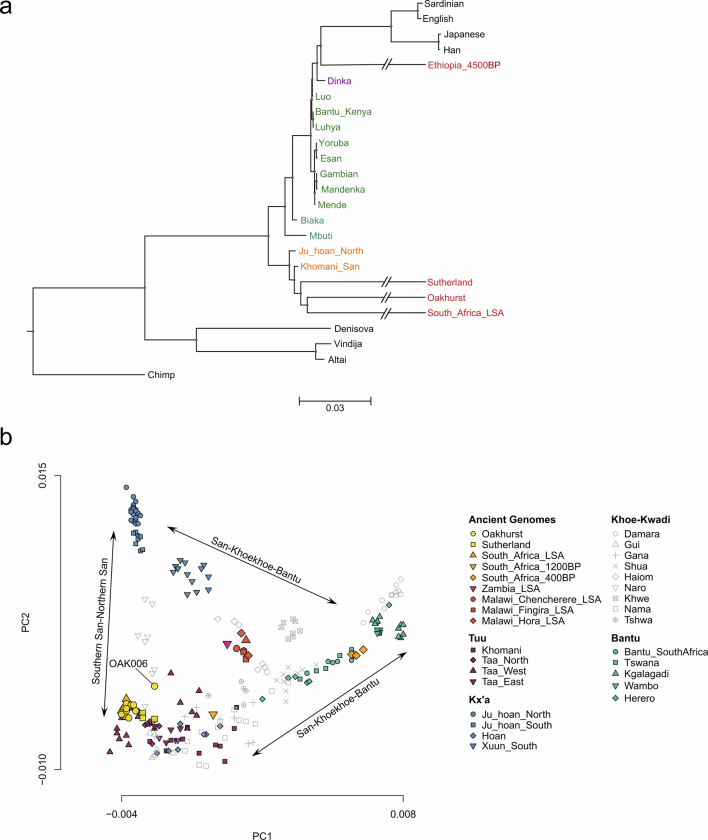
Genetic affinities between ancient and present-day southern Africans. **a**) Maximum likelihood tree, generated using TreeMix (see Methods) of genome sequences from present-day and ancient populations, excluding populations with evidence of asymmetrical allele sharing with non-Africans indicative of recent gene flow. Branches of ancient individuals/groups are truncated for better readability. **b**) Alternative PCA computed using 22 present-day populations from southern Africa and 597,000 SNPs. This PCA drops two populations from the 24 shown in Fig. 1b, for the benefit of using a larger number of SNPs. The arrows indicate the three distinct clines of genetic variation between Bantu groups and Northern or Southern San, respectively. Khoekhoe populations cluster in between the three poles.

**Extended Data Fig. 2:**
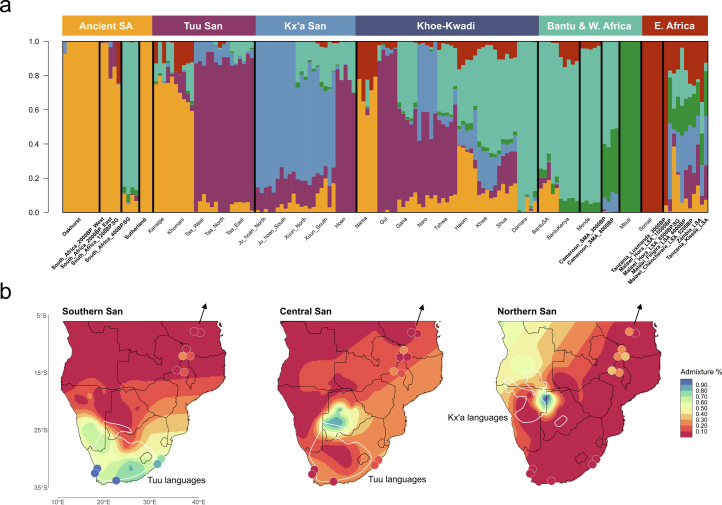
Sample age-informed ancestry decomposition. **a**) Clustering of 162 ancient and present-day sub-Saharan African individuals (~212,000 SNPs) at *K* = 6 using DYSTRUCT. Major groups are indicated on top of the bar plots based on chronological, geographical and linguistic classifications. **b**) Kriging interpolated distributions of southern (orange component), central (magenta component) and northern (blue component) San ancestry in present-day populations. Proportions for ancient LSA individuals are indicated as dots. White outlines indicate the present-day distributions of the Tuu (left and centre) and Kx’a (right) language groups.

To place the nine individuals from Oakhurst into a pan-African evolutionary context, we first constructed a population tree based on allele frequencies in ancient and present-day populations, using TreeMix^25^ (Methods). Similar to contemporary San individuals^1,2,7^, the nine Oakhurst individuals diverge basal to all other human lineages (Extended Data Fig. 1a). To investigate their genetic ancestry in detail, we performed principal component analysis (PCA) on a set of 24 contemporary San, Khoekhoe and Bantu-speaking populations from Namibia, Botswana and South Africa and projected new and published ancient genomes onto the first two principal components (Fig. 1 and Supplementary Tables 26 and 27). In line with previous analysis of San and Khoekhoe fine-scale population structure, we observed marked genetic differentiation between San and Khoe populations along PC2, reflecting the geographic separation between groups living north and south of the Kalahari Desert^7,8,26–29^. In total, we observe three principal clusters with the Kx`a-speaking Ju|’Hoan (genetic group label in figures and tables: Ju_hoan) and !Xuun (Xuun) representing the northern San ancestry component, the Khoe-Kwadi-speaking Nama as well as Tuu-speaking ‡Khomani (Khomani) and Karretjiemense (self-identification of these San descendants from the Karoo region of South Africa, the Afrikaans term translates to ‘the people of the cart’) (Karretjie) forming the southern San ancestry component and the Tuu-speaking Taa, Kx`a-speaking ǂHoan (Hoan) and Khoe-Kwadi-speaking Gǀui (Gui) and Gǁana (Gana) corresponding to the central San ancestry component. In this context, eight of the Oakhurst individuals cluster closely together with four previously published LSA hunter-gatherers from South Africa^1,2^ within the diversity of the southern San and Khoekhoe cluster (Fig. 1b). We also considered a slightly different PCA with a larger SNP overlap among fewer analysed populations (Extended Data Fig. 1b), which proves useful for specific signals. Here, we see that the oldest individual in our dataset, OAK006, which dates between 9,900 and 10,500 cal BP (95% confidence interval (CI)), is slightly shifted in the direction of the northern San cluster (Extended Data Fig. 1b). Unsupervised ancestry decomposition using DYSTRUCT^30^ (Extended Data Fig. 2) shows overall a similar pattern as PCA (with *K* = 6 clusters): present-day southern San and Khoekhoe are assigned the same major ancestral component (shown in orange), which is maximized in the LSA individuals from Oakhurst, St. Helena, Faraoskop and Ballito Bay. In contrast, northern San exhibit a different component (shown in blue) that is maximized in northern Ju|’Hoan. Finally, a third component (shown in magenta) is maximized in Taa groups and also represents the largest San ancestry component in most remaining Khoe-Kwadi populations such as the ǂHoan, Gǀui, Gǁana and Tshwa (Extended Data Fig. 2).

**Extended Data Fig. 3:**
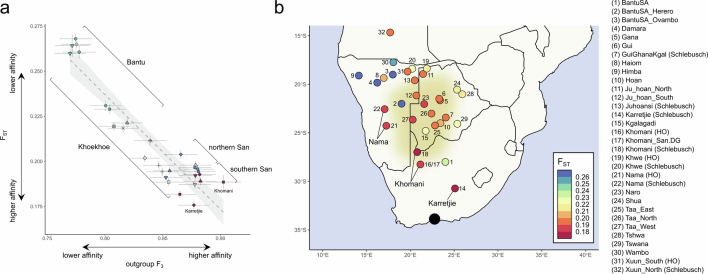
Genetic affinities of Oakhurst individuals to present-day southern Africans in F-statistics. **a)** Scatter plot of group-based jackknife point estimates from F_ST_ (Y axis) and outgroup F_3_-statistics of the form F_3_(Archaic; Oakhurst, X), where X represents the present-day populations (*n* = 32). Error bars represent 2 standard errors. The error band indicates the 95% confidence interval of the linear regression. Symbols and colours correspond to Fig. 1. Data can be found in Supplementary Table 2 & 3. **b**) F_ST_ distances visualized on a map of southern Africa. The populations exhibiting the highest genetic affinity to Oakhurst are highlighted. The location of Oakhurst is plotted in black. The beige-coloured region is the Kalahari semi-desert. Data can be found in Supplementary Table 3.

**Extended Data Fig. 4:**
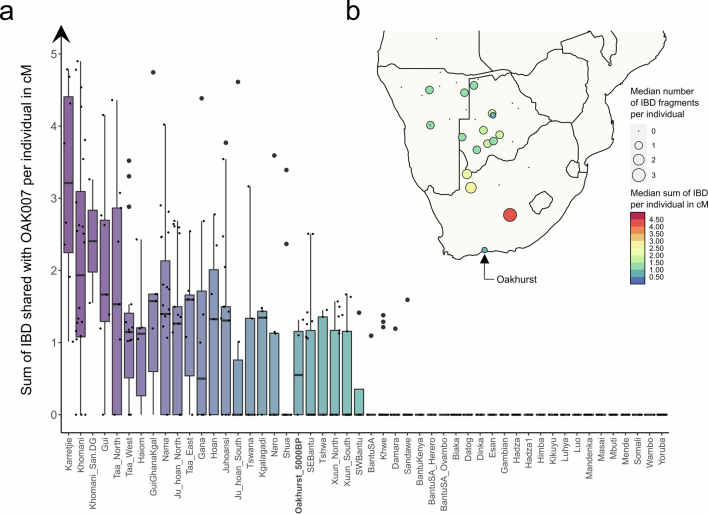
Identity-by-descent segment sharing between imputed Oakhurst genomes and present-day sub-Saharan Africans. **a)** Length of IBD segments shared between OAK007 and present-day Africans. The bounds of the box represent the 25^th^ and 75^th^ percentile, the centre represents the median, and Whiskers represent the smallest value greater than the 25^th^ Percentile minus 1.5 times the interquartile range and largest value less than the 75^th^ Percentile plus 1.5 times the interquartile range of the data, respectively. Outliers present the minimum and maximum values in the data and are depicted as large dots. Each dot corresponds to the summed IBD for a unique pair of OAK007 and an individual from the respective population (*n* = 31). The boxplots are ordered according to the averaged length of IBD segments shared between all individuals of a population and OAK007 (including pairs with no IBD sharing). Populations on the left feature the longest mean IBD segment sharing with OAK007, populations on the right the shortest. Lengths of segments are given in centiMorgan (cM). Data can be found in Supplementary Table 24. **b**) Median IBD sharing between OAK007 and present-day populations visualized on a map of southern Africa. The colour of each dot corresponds to the median length of summed IBD segments shared with OAK007 per individual, the size corresponds to the median number of IBD segments shared per individual.

To quantitatively test whether the observations from the PCA and ancestry clustering are consistent with patterns of shared genetic drift, we compute outgroup *F*_3_-statistics of the form *F*_3_(Archaic; Oakhurst, Test) between the Oakhurst individuals and present-day San and Khoe populations (Extended Data Fig. 3a and Supplementary Table 2). We also calculate the fixation index (*F*_ST_) by pairs and compare the two measures (Extended Data Fig. 3a and Supplementary Table 3). Although outgroup *F*_3_ and *F*_ST_ point estimates are significantly associated (Pearson’s correlation; *t* = −13.472, d.f. = 27, *P* = 1.683 × 10^−13^, r = −0.933) (Extended Data Fig. 3a), the outgroup *F*_3_ signal is mostly correlated with the proportion of indigenous San ancestry (measured using qpAdm; see Methods and analysis around Figs. 3 and 4) in the present-day populations (Pearson’s correlation; *t* = 16.67, d.f. = 9, *P* = 8.478 × 10^−13^, r = 0.968). *F*_ST_ appears less affected by varying percentages of non-San ancestry (evidenced by a reduced correlation between *F*_ST_ and San ancestry; Pearson’s correlation; *t* = −6.252, d.f. = 19, *P* = 5.278 × 10^−6^, r = −0.82) and is consequently able to detect subtle population structure that was obscured by later admixture events. We find the highest genetic affinity between Oakhurst and groups of the southern San cluster, namely the Karretjiemense, ‡Khomani and Nama (Extended Data Fig. 3b). In general, *F*_ST_ between the Oakhurst individuals and present-day San and Khoe-Kwadi-speaking groups is strongly correlated with latitude (Pearson’s correlation; *t* = 2.828, d.f. = 23, *P* = 0.009528, r = 0.508), demonstrating that San and Khoekhoe groups who live closer to Oakhurst rockshelter are still today more closely related to its LSA inhabitants than groups from further north. This is furthermore supported by the sharing of identity-by-descent (IBD) segments between the most recent individual, OAK007 (dating to ~1,344 cal bp; 1,400-1,300 cal bp 95% CI) and present-day southern Africans. On average, OAK007 shares more and longer IBD segments with the Karretjiemense and ‡Khomani than with any other tested population, demonstrating direct genetic relatedness between the ancient hunter-gatherer and modern San and Khoe groups from South Africa (Extended Data Fig. 4 and Supplementary Table 24).

### Genomic continuity since the early Holocene

**Fig. 2:**
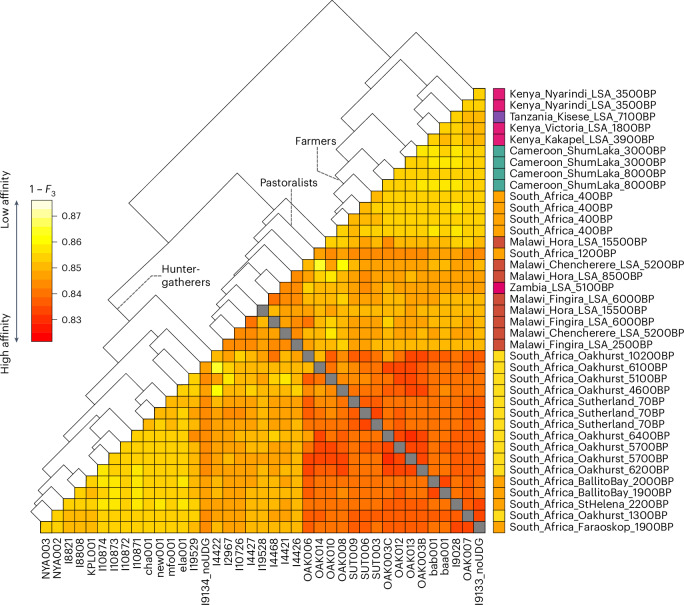
Genetic affinities among LSA individuals from sub-Saharan Africa. Shown is a heat-map matrix of pairwise outgroup *F*_3_-statistics of the form *F*_3_(X, Y; Chimp). Hierarchical cluster analysis applying Ward’s minimum variance method to the rows is added as a dendrogram. Data can be found in Supplementary Table 4.

**Extended Data Fig. 5:**
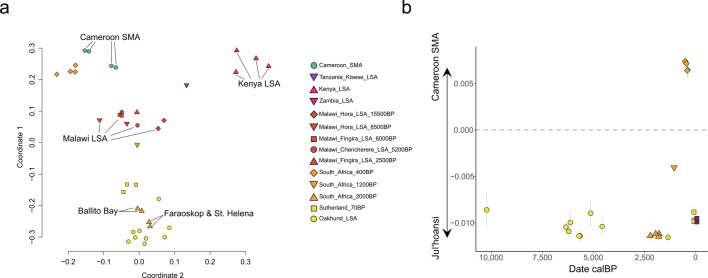
Population structure in ancient Africa. **a)** MDS plot of the pairwise F_3_-matrix of the form F_3_(Chimp; X, Y) (shown in Fig. 2) transformed into distances using the formula 1 − F_3_. Data can be found in Supplementary Table 4. **b**) Individual F_4_-statistics of the form F_4_(Archaic, Test; Ju_hoan_North, Cameroon_SMA) through time for 21 ancient and 2 current-day Khomani genomes from South Africa. Error bars represent 2 standard errors. Data can be found in Supplementary Table 5.

We then proceeded to investigate individual changes in ancestry through time. First, we assessed the extent of genetic similarity between the Oakhurst individuals and previously published prehistoric genomes from South Africa, Cameroon^31^, Kenya^32,33^, Malawi^2,34^, Tanzania^34^ and Zambia^34^ by means of outgroup *F*_3_-statistics (Supplementary Table 4). All LSA genomes from South Africa are more like one another than any other tested prehistoric ancient African (Fig. 2). Yet, some fine-scale population stratification is evident, with the most recent sample OAK007 clustering together with two 2,000-year-old hunter-gatherers from St. Helena and Faraoskop. Together these samples form a sister clade with two contemporaneous samples from Ballito Bay, located in KwaZulu-Natal on the eastern coast of South Africa, within the diversity of the older Oakhurst samples. Visualizing the transformed pairwise-distance *F*_3_-matrix through multidimensional scaling, we find the Oakhurst individuals older than 1,300 cal bp shifted along coordinate 1 away from the younger LSA genomes and three historical San samples from Sutherland, Western Cape province (Extended Data Fig. 5a). On the other hand, consistent with the affinities detected in PCA and DYSTRUCT analysis, the genomes of a 1,200-year-old pastoralist and four Iron Age farmers from South Africa cluster in the diversity of LSA genomes from Malawi and Cameroon, respectively, highlighting the impact of admixture events after 1,300 cal bp that contributed varying fractions of non-San ancestry to all populations in southern Africa^1,2,7,8^ (Fig. 2).

**Fig. 3:**
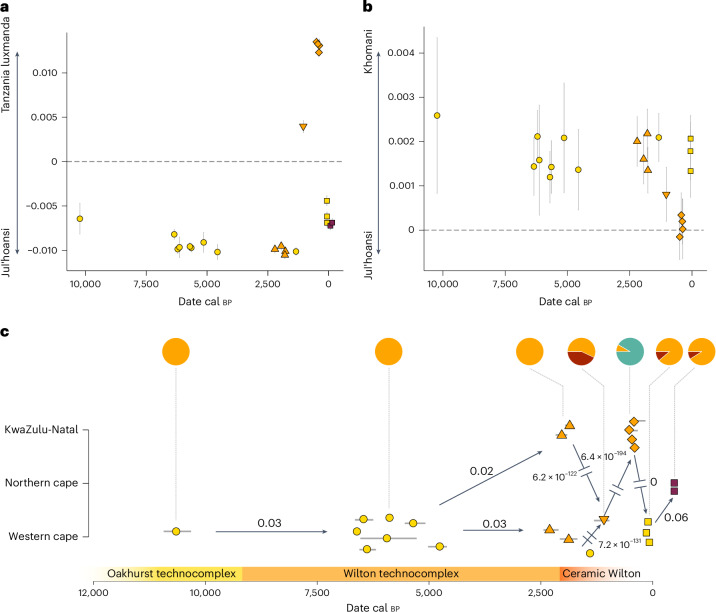
Long-term genetic continuity and abrupt disruption in South Africa. **a**, Individual *F*_4_-statistics of the form *F*_4_(Archaic, Test; Ju_hoan_North, Tanzania_Luxmanda_3000BP) through time for 21 ancient and 2 current-day Khomani genomes from South Africa. Error bars represent 2 s.e. Data can be found in Supplementary Tables 6 and 7. **b**, Individual *F*_4_-statistics of the form *F*_4_(Archaic, Test; Ju_hoan_North, Khomani_San) through time for 21 ancient genomes from South Africa. **c**, Overview about population genetic changes in South Africa from 10,000 cal bp to the present-day. Arrows indicate *P* values from generalized-likelihood ratio tests implemented in qpWave testing for genetic continuity between temporally preceding and succeeding groups in the Northern Cape, Western Cape and KwaZulu-Natal, respectively. Discontinuities are explicitly marked as interrupted arrows. Pie charts depict the ancestry composition for each group derived from qpAdm modelling. Symbols and colours correspond to Fig. 1. Data can be found in Supplementary Tables 8 and 9.

To test whether the Oakhurst individuals already exhibit subtle excess affinity to non-San ancestries, we calculated *F*_4_-statistics^35^ of the form *F*_4_(Archaic, Test; Ju_hoan_North, Tanzania_Luxmanda_3000BP) (Fig. 3a and Supplementary Table 6) and *F*_4_(Archaic, Test; Ju_hoan_North, Cameroon_SMA) (Extended Data Fig. 5b and Supplementary Table 5). We find that none of the Oakhurst individuals, including the most recent individual OAK007, shares significantly more genetic drift with Tanzania_Luxmanda_3000BP (associated with East African pastoralist ancestry) or Cameroon_SMA (representing Central and Western African ancestry) than the published LSA genomes, providing an early bound for the date of the arrival of non-San ancestry at the southern coast only after 1,300 cal bp. For all LSA samples from South Africa, we observe significantly higher affinity (*Z* > 3) to present-day ‡Khomani than to Ju|’Hoan, confirming an old split age for the northern and southern San ancestries before 20,000 bp (refs. ^7,8,27,36^) and, thus, before the lake Makgadikgadi palaeo-wetland dried up^29,37^ (Fig. 3b and Supplementary Table 7).

To evaluate whether chronological groups in South Africa were consistent in sharing the same genetic make-up as the preceding and succeeding populations, we used qpWave^38–40^, a generalization of *F*_4_-statistics, testing for significant evidence of continuity (that is, we tested whether they were consistent with forming a clade at *P* > 0.01) (Supplementary Table 8). We find that groups of individuals from Oakhurst dating to 10,000 cal bp and 6,000 to 4,000 cal bp as well as groups from the western and eastern coast (St. Helena, Faraoskop, OAK007 and Ballito Bay, respectively; dating between 2,200 and 1,300 cal bp) were all genetically indistinguishable (Fig. 3c). On the other hand, we observe significant discontinuity between 1,300 and 1,200 cal bp, as well as between 1,200 and 400 cal bp, consistent with the independent arrivals of non-San East African pastoralist and West African farmer ancestry in South Africa (Fig. 3c). To assess these demographic changes quantitatively, we used qpAdm^39,40^ to successively model these groups as mixtures between local LSA, pastoralist and farmer ancestry components (Methods; Supplementary Table 9). We find no evidence of West African ancestry within the three Sutherland individuals^41^ (dating to the second half of the nineteenth century), yet, we detect small amounts of Tanzania_Luxmanda_3000BP-related ancestry (11% ± 0.9%) comparable to the proportions measured in present-day ‡Khomani from the Northern Cape Province (9% ± 1%) (Fig. 3c).

**Extended Data Fig. 6:**
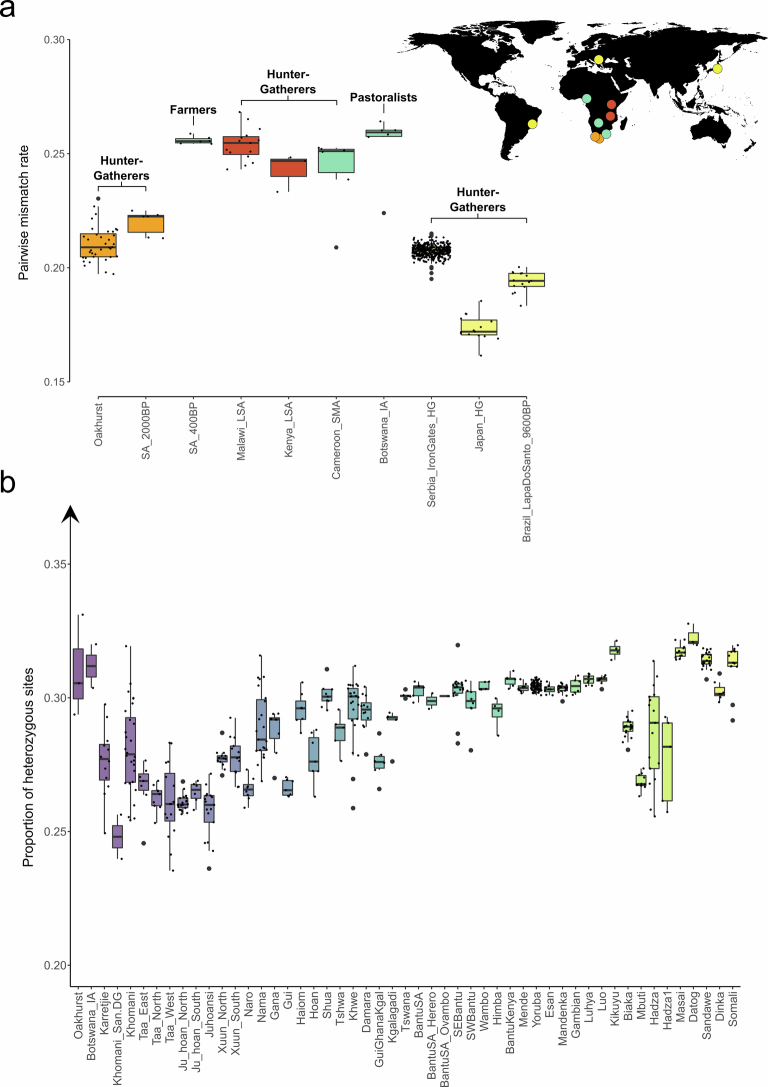
Genetic diversity in ancient and present-day sub-Saharan Africans. **a**) Conditional nucleotide diversity (measured as the intra-population pairwise mismatch rate) in ancient pseudo-haploid populations (*n* = 10). Each dot depicts the pairwise mismatch rate between a pair of individuals from the same group. The bounds of the box represent the 25th and 75th percentile, the centre represents the median, and Whiskers represent the smallest value greater than the 25th Percentile minus 1.5 times the interquartile range and largest value less than the 75th Percentile plus 1.5 times the interquartile range, respectively. Outliers present the minimum and maximum values in the data and are depicted as large dots. The geographical origin of each ancient group is indicated on the world map. **b**) Average heterozygosity in 5 imputed ancient genomes from South Africa and Botswana and 561 individuals from 49 present-day African populations. Each dot corresponds to the fraction of heterozygous sites over the total number of genotyped sites across 22 autosomes. The bounds of the box represent the 25th and 75th percentile, the centre represents the median, and Whiskers represent the smallest value greater than the 25th Percentile minus 1.5 times the interquartile range and largest value less than the 75th Percentile plus 1.5 times the interquartile range, respectively. Outliers present the minimum and maximum values in the data and are depicted as large dots. Data can be found in Supplementary Table 25.

Overall, these observations indicate that between 10,000 and 1,300 cal bp, no ancestry from outside present-day South Africa arrived at Oakhurst rockshelter, demonstrating a remarkable degree of relative genetic continuity over a time range of nearly 9,000 years. Such a demographic pattern is exceptional in the global archaeogenetic record, yet, the Oakhurst samples do not exhibit signs of genetic isolation. While the conditional nucleotide diversity (CND; Methods) of the Oakhurst individuals is lower than in LSA populations from Malawi, Kenya and Cameroon, it is comparable to the diversity measured in the published hunter-gatherers from the Western Cape and KwaZulu-Natal and higher than the CND in ancient hunter-gatherers from Serbia^42^, Japan^43^ or Brazil^44^ (Extended Data Fig. 6a). Furthermore, we find the average heterozygosity levels within the three highest-coverage individuals (OAK007, OAK012 and OAK013) to be higher than in most present-day San and Khoe populations, disagreeing with a model of prolonged genetic isolation, yet supporting recent findings of a continuous, substantial reduction of effective population size in southern San and Khoe after 1,300 bp (ref. ^45^) (Extended Data Fig. 6b and Supplementary Table 25).

### Demographic changes in the last 2,000 years

**Extended Data Fig. 7:**
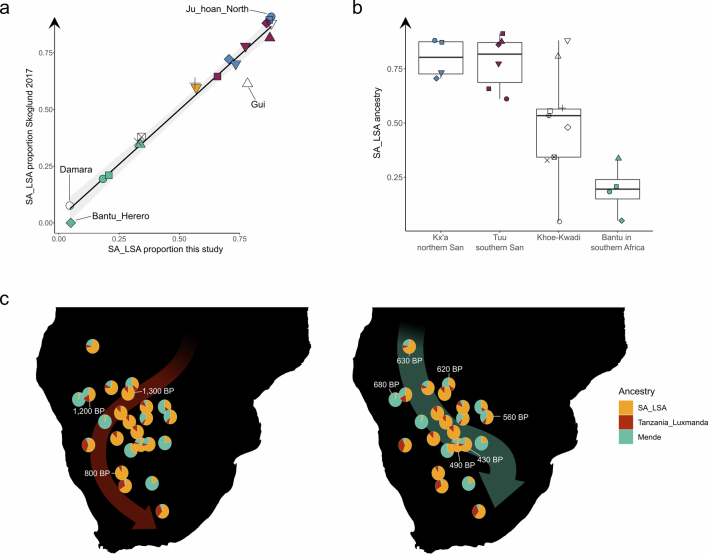
Ancestry composition of present-day San, Khoe and Bantu populations. **a**) Scatter plot of the proportions of SA_LSA ancestry measured by Skoglund et al. 2017 (represented by the St. Helena and Faraoskop genomes) and this study (represented by the Oakhurst, St. Helena, Faraoskop and Ballito Bay genomes) in present-day San, Khoe and Bantu-spreaking groups (*n* = 19) using qpAdm. The error band indicates the 95% confidence interval of the linear regression. Symbols and colours correspond to Fig. 1. **b**) Fractions of SA_LSA ancestry in San, Khoe and Bantu groups stratified by language affiliation. The bounds of the box represent the 25th and 75th percentile, the centre represents the median, and Whiskers represent the smallest value greater than the 25th Percentile minus 1.5 times the interquartile range and largest value less than the 75th Percentile plus 1.5 times the interquartile range, respectively. **c**) Ancestry compositions retrieved from 2- and 3-way qpAdm admixture modeling of present-day San, Khoe and Bantu groups (*n* = 23) visualized on a map of southern Africa. Mean admixture dates from DATES analysis are indicated for selected populations. On the left for populations admixed between SA_LSA and Tanzania_Luxmanda_3000BP and on the right for populations admixed between SA_LSA and Mende. Arrows on maps indicate a general direction of influences rather than discrete routes of migration. Data can be found in Supplementary Table 10-12.

We use the increased availability of LSA data to quantify and characterize the demography of transitions in southern Africa during the last 2,000 years. Yet, these admixture events are challenging to reconstruct because of additional gene flow from at least two immigrant populations during prehistoric times^1,2,7,8,27,46–49^ and additional inter- and intra-continental admixture following European settlement from the 1650s onwards^46,50–52^. This complex history hinders inferences about the timing and mode (for example, involving sex bias) of admixture events. To circumvent these issues, we focused on groups with only two of the various ancestries present in the region today and compared the resulting patterns to identify putative trajectories of non-LSA ancestries through Southern Africa. Specifically, we used qpAdm to test 1-source, 2-source or 3-source models (excluding individuals with European admixture for now) for present-day San, Khoe and Bantu-speaking populations. As sources, we used (1) Stone Age hunter-gatherers from South Africa (SA_LSA), (2) Tanzanzia_Luxmanda_3000BP and (3) present-day Mende, reflecting the local LSA, pastoralist and farming ancestries^2^, respectively (Methods; Extended Data Fig. 7 and Supplementary Tables 10–12).

**Extended Data Fig. 8:**
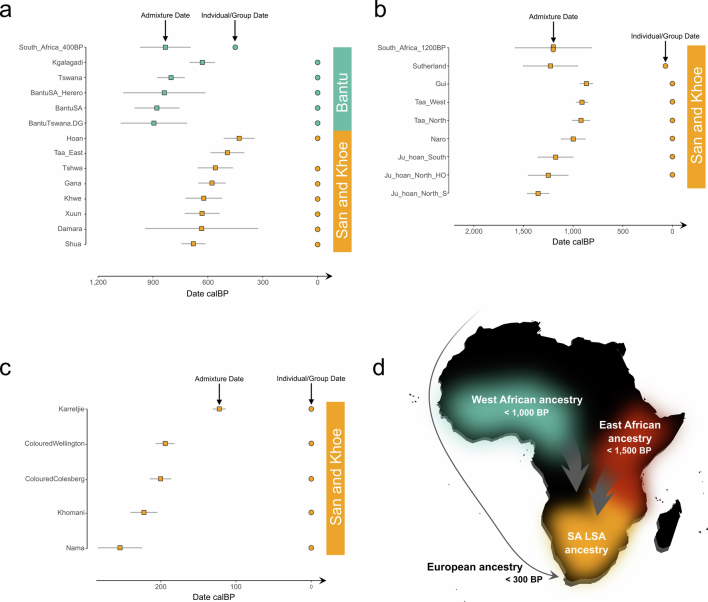
Admixture dates in ancient and present-day San, Khoe and Bantu populations. **a**) Admixture date point estimates for the admixture (squares) between SA_LSA and Mende in groups with primarily West African-related admixture (based on qpAdm) (*n* = 9). For ancient individuals, the mean radiocarbon date is indicated (circles). Error bars represent 1 standard error. **b**) Admixture date point estimates for the admixture (squares) between SA_LSA and Tanzania_Luxmanda_3000BP in groups with primarily East African-related admixture (based on qpAdm) (*n* = 14). Error bars represent 1 standard error. **c**) Admixture date point estimates for the admixture (squares) between SA_LSA and English in groups with European-related ancestry (*n* = 5). Error bars represent 1 standard error. **d**) Schematic representation of the geographic origin and potential admixture date of non-Southern African ancestry found in present-day San and Khoe populations. Data can be found in Supplementary Table 13-16.

On the basis of the estimated admixture proportions in the best-fitting model using the lowest number of sources, we grouped populations into primarily West African- or East African-admixed categories, excluding ambiguous cases with both non-San ancestries being present substantially (Supplementary Tables 13–16). Within these categories, we computed admixture dates for the West African component (Extended Data Fig. 8a and Supplementary Table 14) and the East African component (Extended Data Fig. 8b and Supplementary Table 13) in the relevant target groups. We find East African, Tanzania_Luxmanda-related ancestry gene flow into San and Khoe populations to be consistently older than the gene flow from West African-related groups. For the Tanzania_Luxmanda-related gene flow, we identify a mean admixture date of 1,068 bp among San and Khoe populations, agreeing with the observed East African ancestry in the 1,200-year-old pastoralist from Kasteelberg and the admixture date estimated for the nineteenth century Sutherland samples (1,228 ± 278 bp). In contrast, West African admixture in San, Khoe and Bantu groups is dated consistently younger than the Tanzania_Luxmanda-related ancestry gene flow and also exhibits a difference between Bantu and San/Khoe. Specifically, the mean date for admixture of West African ancestry among Bantu-speaking populations in southern Africa (for example, Herero, Tswana and Kgalagadi) is estimated at 808 bp on average, remarkably agreeing with the dating of that admixture in the 400 cal bp Iron Age farmers from KwaZulu-Natal (832 ± 139 bp). In contrast, the estimated date of West African ancestry in San and Khoe groups is more recent (578 bp). This discrepancy might suggest successive waves of Bantu immigration^53^ or continuing gene flow from Bantu-related groups into San and Khoe populations after the initial admixture event that contributed San and Khoe ancestry to Bantu-speaking groups (Extended Data Fig. 8).

**Fig. 4:**
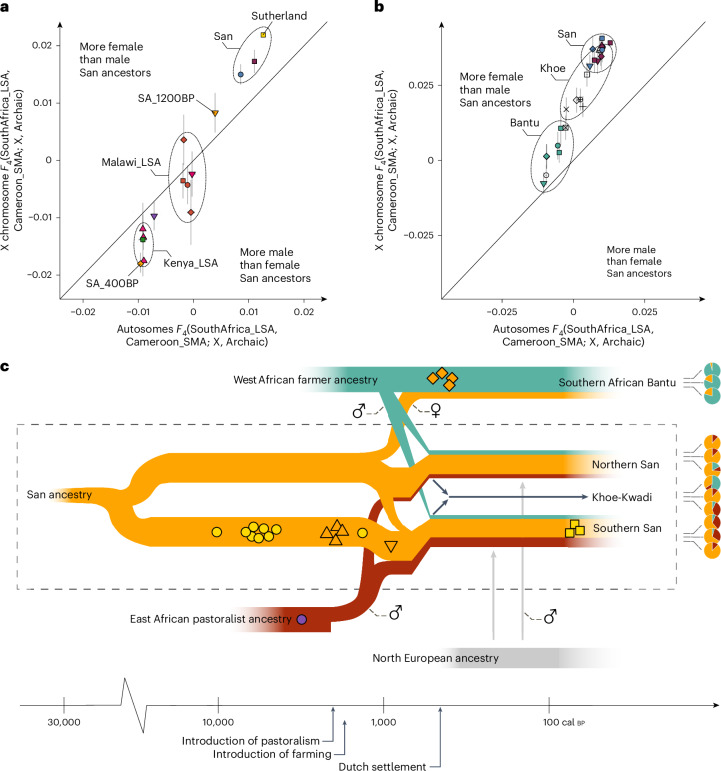
Demographic changes in the San and Khoe populations of southern Africa. **a**, Scatter plot of group-based jackknife point estimates for *F*_4_-statistics of the form *F*_4_(Archaic, X; Cameroon_LSA, SA_LSA) on loci of the X chromosome (*y* axis) and the autosomes (*x* axis), where X represents the ancient genomes (*n* = 17) and two present-day San populations (Khomani_San and Ju_Hoan_North). Error bars, 1 s.e. Data can be found in Supplementary Table 18. **b**, As **a** but for present-day San, Khoe and Bantu-speaking populations (*n* = 25). Error bars, 1 s.e. Data can be found in Supplementary Table 17. **c**, Summary of the inferred population history of the San and Khoe in southern Africa. Sex symbols indicate male- and female-biased reproduction. Note that pastoralism and farming both appeared in present-day South Africa at about the same time, 2,000 years ago. Symbols and colours correspond to Fig. 1.

For the mode of interaction between locals and newcomers, we find evidence for sex bias in most present-day San, Khoe and Bantu populations, with stronger signals in the San and Khoe populations compared to the Bantu-speaking groups (Fig. 4b and Supplementary Table 17). In general, both San and Khoe as well as Bantu groups exhibit significantly more SA_LSA ancestry on the X chromosome than the autosomes and (congruently) share more drift with SA_LSA on the X chromosome than on the autosomes (Fig. 4b). This suggests that substantially more female than male San ancestors were involved in the admixture events following the spread of East African pastoralist and West African farmer ancestry, which is consistent with previous studies of uniparental and genome-wide markers^19,20,22,28,29,47,48,54–57^. Assuming a single admixture event (using the dates obtained from DATES analysis), we explicitly compared autosome to X chromosome ancestry to determine female (*sf*) and male contributions (*sm*) to the gene pools of selected Khoe and Bantu populations, using a previously described method^58,59^ (Supplementary Table 20). For the Damara, we estimate that for each San man ∼1.4 San women contributed to the gene pool, for the ǂHoan ∼2.28 San women per San man, for the Shua ∼4, for the Haiǁom (Haiom) ∼5.2 and for South African Bantu ∼2.1 San women per San man.

Although this signal of female-biased admixture is also evident in the historical Sutherland genomes and the 1,200-year-old pastoralist from Kasteelberg, we observe that the four Iron Age KwaZulu-Natal samples (SA_400BP) share more drift with SA_LSA on the autosomes than on the X chromosome, indicative of more male than female San ancestors (Fig. 4a and Supplementary Tables 18 and 19). This contradicts the pattern observed in most present-day Bantu or San and Khoe groups in South Africa and Botswana and might be related to changes in interaction between Bantu and San/Khoe groups after 400 bp. Overall, our results show that, despite an overarching trend of female-biased gene flow from San and Khoe populations into Bantu-speaking groups, the modes of interaction and reproduction were strongly influenced by locally and temporally defined factors after the initial arrival of the first farmers^47^.

**Extended Data Fig. 9:**
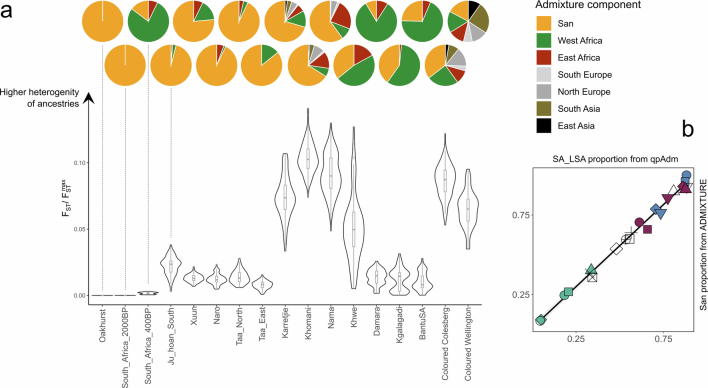
Variability in admixture proportions among ancient and present-day San, Khoe, Bantu and Coloured populations. **a**) Pie charts depict the averaged ancestry composition for each group derived from supervised ADMIXTURE modeling at *K* = 7. Box- and violin plots depict the Bootstrap (*n* = 1000) distributions of the ancestry variability measure, F_ST_/F_ST_^max^, obtained from FStruct (methods) calculated on the Q matrix from supervised ADMIXTURE for each population. The bounds of the box represent the 25th and 75th percentile, the centre represents the median, and Whiskers represent the smallest value greater than the 25th Percentile minus 1.5 times the interquartile range and largest value less than the 75th Percentile plus 1.5 times the interquartile range, respectively. **b**) Scatter plot of the proportions of SA_LSA ancestry measured using qpAdm and the San proportion measured using supervised ADMIXTURE in present-day San, Khoe and Bantu-spreaking groups. Both estimates are significantly correlated (Pearson's correlation; *t* = 49.826, *df* = 20, *p* = 2.2e-16, *cor* = 0.996). The error band indicates the 95% confidence interval of the linear regression. Symbols and colours correspond to Fig. 1. Data can be found in Supplementary Table 21 & 22.

Finally, we detect a comparatively recent admixture date corresponding to male-biased^28,46,50–52^ gene flow from Europe into San/Khoe and mixed^60^ groups from Colesberg and Wellington^7^ (Supplementary Tables 21–23). This ancestry is best approximated by northwestern Europeans, as shown by admixture *F*_3_-statistics of the form *F*_3_(Test, Sutherland, Target) (Supplementary Table 23). For instance, for South Africans of mixed ancestry from Colesberg, we observed that *F*_3_ values among 40 West Eurasian populations are minimized for Irish, Icelandic and Norwegian people, followed by English (all *Z* < −69). We date the arrival of northern European ancestry among these populations to 199 yr bp (Extended Data Fig. 8 and Supplementary Table 15), postdating the settlement of South Africa by Dutch and British immigrants from the mid-1600s onwards, a development that led ultimately to the demise of most San and Khoe genetic, linguistic and cultural diversity in the region^61^ and lastingly affected the demographic trajectories in southern Africa^62^ (Fig. 4c). While population structure in South Africa partly collapsed, new extracontinent ancestries were introduced to the region, increasing the heterogeneity of the admixture landscape. To quantitatively estimate this influx from outside Africa and its impact on genetic diversity, we decomposed admixture sources using a supervised clustering approach implemented in the software ADMIXTURE^63^ (Extended Data Fig. 9 and Supplementary Tables 21 and 22). For example, in South Africans of mixed ancestry from Colesberg, we observe on average 24.4% ± 3.2% South Asian ancestry and 2.8% ± 0.5% East Asian ancestry besides 8.2% ± 1.4% North European ancestry, yet only 35.5% ± 2.9% San-related ancestry. Besides South Africans of mixed ancestry, we furthermore detect European ancestry in the Karretjiemense (5.61%), ‡Khomani (9.45%) and Nama (6.83%), indicating that the southern San and Khoe were especially affected by admixture with European sources. Using the output from ADMIXTURE, we proceeded to measure the variability of ancestry components in the southern African groups via FSTruct^64^. We find that the relative levels of variation among southern San, namely the Karretjiemense, ‡Khomani and Nama are significantly higher than in any other present-day San or Khoe population because of the frequent presence of variable European ancestry components, comparable to the variability measured in South Africans of mixed ancestry from Colesberg and Wellington (Supplementary Tables 21 and 22 and Extended Data Fig. 9). This heterogeneity in non-Southern African admixture across individuals obscures the high genetic affinity to the ancient Oakhurst samples, as measured using *F*_ST_ and IBD metrics and highlights the necessity of further sampling of local communities to adequately assess the effect of non-southern African admixture on the current genetic landscape of San populations in southernmost Africa. On the other hand, northern and central San feature significantly lower variability, which is similar to the diversity observed in neighbouring Bantu-speaking people, who also do not exhibit substantial proportions of non-African ancestry (Extended Data Fig. 9).

## Discussion

The question of population continuity or discontinuity during the LSA of southern Africa has been the focus of anthropological research for well over a century. Archaeogenetic research of the last two decades has revealed that the Holocene demographic histories of Stone Age Europe^39,65–71^, Asia^43,72–77^ and North Africa^78–80^ were characterized by several episodes of large-scale migrations, either in the form of admixture with newcomers or by total replacement of the established inhabitants. While these biological transformations modified the genetic make-up of the local populations, they were also vectors for technological innovation, such as the introduction of new technologies, raw material uses or subsistence strategies. In contrast, for South Africa, we demonstrate that the local gene pool was characterized by a prolonged period of genetic continuity with no (detectable) gene flow from outside southern Africa. The earliest individual in our study that yielded aDNA showed a genetic make-up indistinguishable from the later inhabitants of Oakhurst rockshelter, suggesting that this local ‘southern’ San gene pool was formed more than 10,000 years ago and remained isolated from admixture with neighbouring ‘central’ and ‘northern’ San populations^7,8,26–29^ or with more distant sources to the northeast, which admixed with San groups in Malawi and Tanzania^2^.

Consequently, the sequence of cultural change at Oakhurst, for example from the Oakhurst to Wilton technocomplexes^6,9,14,15^, appears to result from local development initiated by the indigenous inhabitants^14^, highlighting the role of in situ innovations followed by acculturation. Our data also demonstrate that subtle fluctuations in the craniofacial size of South African LSA coastal inhabitants^81,82^ (for example, between 4,000 and 3,000 bp) were not the product of genetic discontinuity but probably related to changes in environmental factors or population size^81–83^. Yet, we highlight that the inhabitants of Oakhurst were not a small, bottlenecked population. Genomic measurements of diversity indicate a degree of genetic variation comparable to other African hunter-gatherer populations and higher than Stone Age foragers from Europe or America. Furthermore, the current resolution of our methods and limited reference dataset in sub-Saharan Africa restricts our ability to detect subtle changes in group size or small-scale immigration of people from within southern Africa. However, our data are congruent with a population in reproductive isolation from other San (and non-San) populations over the whole period of occupation of the site.

This period of ~9,000 years of genetic continuity ends rather abruptly in the migration events which introduced East and West African-related ancestry to South Africa, accompanied by the spread of herding and farming^1,2,7,8^. On the basis of present available data, it appears that non-southern African ancestry reached the southernmost parts of South Africa only after 1,300 cal bp. There is, however, abundant archaeological evidence of marked changes in subsistence and settlement patterns among coastal and near-coastal communities in this region from ~2,000 cal bp. These changes have previously been interpreted as resulting from the disruption of hunter-gatherer communities by the emergence of herding^84–87^. Notably, a similar temporal discrepancy was observed during the Mesolithic–Neolithic translation in Europe, where the admixture between hunter-gatherers and incoming farmers postdates the emergence of agriculture by almost 2,000 years (ref. ^88^). This indicates that hunter-gatherers and farmers resided in close geographic proximity for a considerable time before mixing^88^ and demonstrates that migration can precede any subsequent population admixture substantially. Alternatively, the practice of pastoralism may have spread to Southern Africa through a process of cultural diffusion in advance of substantial population expansion^89,90^, explaining the absence of any East African-related ancestry in South Africa before 1,300 cal bp.

Yet, the events of the last 1,300 years had a substantial impact on the local gene pool of South Africa. Today, all San and Khoe populations are admixed with one or both of East African Pastoralist and West African Farmer ancestry^1,2,7,8^. The collapse of the LSA population structure was accelerated by the arrival of European settlers in the mid-seventeenth century^62^. Together with the continuous loss of oral traditions, these issues contribute to our poor understanding of the prehistoric southern African population structure. Using allele-frequency and IBD segment-based analyses, we were able to show that the present-day San and Khoe inhabitants of South Africa are, despite recent periods of disruption under Dutch and British rule, still directly related to the ancient Oakhurst individuals of the last 10,000 years. Especially among the ‡Khomani, Karretjiemense and Nama, who belong to the most admixed San/Khoe groups in southern Africa, some individuals still trace most of their ancestry back to these LSA hunter-gatherers. This also applies to the three historic San individuals from Sutherland dating to the late nineteenth century, who show only minor ancestry contribution from outside southern Africa and otherwise close autosomal and mitochondrial similarity to the LSA Oakhurst population^41^, demonstrating that the early Holocene gene pool of the Western Cape persisted in some regions throughout the last 2,000 years without major changes and that in some parts of southern Africa the long-lasting population continuity was not completely disrupted.

## Methods

### Study design and ethics

The human remains from the Oakhurst rockshelter site are housed in the University of Cape Town (UCT) human skeletal repository. The approach for permission to use these samples for aDNA was followed according to ref. ^91^, which included consultation with representatives from the San community in accordance with the South African Heritage Resources Agency and permission from the repository research committee. The Oakhurst samples were approved by the UCT human research ethics committee under ethics no. 715/2017 and Heritage Western Cape permit no. 17071302AS0718E.

The sampling strategy used was twofold: to be minimally invasive and serial sample through time in the occupation of the site. We selected only individuals with loose, broken or previously glued petrous bones so that upon return the samples could be re-glued back to their original state (only featuring a small, unnoticeable sampling hole). Additionally, a single tooth per individual was sampled. For the DNA libraries analysed in this study, 13 individuals were sampled (including 11 petrous bones and 12 teeth). OAK003.B and OAK003.C were initially thought to belong to the same individuals but in fact represent two distinct individuals. These small bone and tooth elements were shipped to Germany for sampling and (after processing) returned to South Africa. Collection of bone powder for aDNA extraction was performed as described in the section on ‘Ancient DNA work’ below.

New radiocarbon dates for this study were measured on the bone and tooth fragments sampled for DNA. These dates were obtained at the Curt-Engelhorn-Center Archaeometry gGmbH, Mannheim, using MICADAS-AMS. Collagen was extracted from the previously sampled bones, purified by ultrafiltration (fraction >30 kDa) and freeze-dried. The ^14^C ages were normalized to δ^13^C = −25‰. The calibration was done using the SHCal20 calibration curve for the Southern Hemisphere^92^.

### Site background

Oakhurst rockshelter (33° 59′ 00″ S–34° 00′ 00″ S and 22° 35′ 00″ E–22° 43′ 00″ E) is important in the history of LSA studies in southern Africa. Excavations by John Goodwin from 1932 to 1935 produced many artefacts and some human skeletons. The site has substantial deposits (>2 m deep) extending over the last 10,000 years. It was one of the first in South Africa to be excavated in accordance with professional standards, with Goodwin and his team carrying out meticulous excavation and detailed recording. In the 1930s, a main goal was a better understanding of the stratigraphic (and thus temporal) relationships between different stone artefact assemblages, seen at that time as different ‘cultures’.

Now that more sites have been excavated, we recognize that the large, relatively unstandardized stone artefacts from the lower part of the Oakhurst sequence form part of a widespread artefact-making tradition in the terminal Pleistocene/early Holocene, extending across South Africa and into Zimbabwe and southern Namibia. Although there are regional variations, these assemblages are sufficiently similar that they are generally grouped as the ‘Oakhurst technocomplex’, acknowledging their early recognition at this site. At Oakhurst, this technocomplex extends through approximately the lower half of the deposits and dates to 9,000–8,000 bp. At about 8,000 bp there was a shift to microlithic (Wilton) assemblages, also very widely distributed across the subcontinent (and into East Africa). Goodwin distinguished ‘Smithfield C’, ‘Wilton’, ‘Developed Wilton’ and ‘Wilton with pottery’ but today we see these as an evolving microlithic tradition. Selection of fine-grained stone raw materials facilitated the making of tiny artefacts, with materials probably sourced over considerable distances. We note that the inhabitants of Oakhurst continued to make microlithic artefacts into the last 2,000 years (when they also made pottery), as seen at the sites of Boomplaas, further inland and Die Kelders, on the coast further to the west. Along most of the southern Cape coast, however, preferences in the last ~3,500 years shifted back to macrolithic artefacts with very little retouch, often made on locally available quartzite. A greater degree of spatial heterogeneity in the last few millennia is consistent with higher population densities and more territorial settlement patterns, as seen amongst hunter-gatherers in coastal and riverine areas elsewhere in the world.

Excavations at Oakhurst were made considerably more challenging by the many burials, with some grave shafts and even graves intersecting others. Some individuals could be recovered in their entirety, sometimes with rich grave goods, for example grave VIa (UCT 204). Others had been dispersed by disturbances in antiquity or by the roots of plants or burrowing animals, making it difficult to assess how many individuals are represented in the remains recovered.

### Ancient DNA work

#### Collection of bone powder

Sampling of 23 bone and teeth samples took place in clean-room facilities dedicated to aDNA work at the Max Planck Institute for Science of Human History in Jena (MPI-SHH). The sampling workflow included documenting and photographing the provided samples. For teeth, we either cut along the cementum/enamel junction and collected powder by drilling into the pulp chamber or accessed the pulp chamber by drilling the tooth transversally. For the petrous bones, we cut the petrous pyramid longitudinally to drill the dense part directly from either side^93^. We collected 28–178 mg of bone or tooth powder per sample for DNA extractions.

For four bone samples, more bone powder was obtained for ^14^C dating at the Curt-Engelhorn-Center Archaeometry gGmbH.

#### DNA extraction

The aDNA was extracted following a modified protocol of ref. ^94^, as described in ref. ^95^, where we replaced the extended-MinElute-column assembly for manual extractions with columns from the Roche High Pure Viral Nucleic Acid Large Volume Kit^96^ and for automated extraction with a protocol that replaced spin columns with silica beads in the purification step^97^.

#### Library construction

We generated 23 double-indexed^98^, double-stranded libraries using 25 µl of DNA extract and following established protocols^99^. We applied the partial UDG (UDG half)^100^ protocol to remove most of the aDNA damage while preserving the characteristic damage pattern in the terminal nucleotides. Additionally, we generated 15 double-indexed single-stranded libraries^101^ using 20 µl of DNA extract and applied no UDG treatment.

#### Shotgun screening, capture and sequencing

Libraries were sequenced inhouse on an Illumina HiSeq 4000 platform to an average depth of 5 million reads and after demultiplexing processed through EAGER^102^. After an initial quality check based on the presence of aDNA damage and endogenous DNA >0.1%, we subsequently selected and enriched 11 double-stranded and 15 single-stranded libraries using in-solution capture probes synthesized by Agilent Technologies for ~1,240k SNPs along the nuclear genome^103^. The captured libraries were sequenced for ~34 million reads on average (minimum, 17 million; maximum, 52 million) using a single end (1 × 75 base pair (bp) reads) configuration. Taking all double- and single-stranded libraries together, we generated 40–139 million reads for the 13 individuals (on average 62 million reads).

### aDNA data processing

#### Read processing and aDNA damage

After demultiplexing based on a unique pair of indexes, raw sequence data were processed using EAGER^102^. This included clipping sequencing adaptors from reads with AdapterRemoval (v.2.3.1)^104^ and mapping of reads with BWA (Burrows–Wheeler Aligner) v.0.7.12 (ref. ^105^) against the human reference genome hg19, with seed length (-l) disabled, maximum number of differences (-n) of 0.01 and a quality filter (-q) of 30. We removed duplicate reads with the same orientation and start and end positions using DeDup v.0.12.2 (ref. ^102^). Terminal base deamination damage calculation was done using mapDamage v.2.0.6 (ref. ^106^), specifying a length (-l) of 100 bp. For the ten libraries that underwent UDG half treatment, we used BamUtil v.1.0.14 (https://genome.sph.umich.edu/wiki/BamUtil:_trimBam) to clip two bases at the start and end of all reads for each sample to remove residual deaminations, thus removing genotyping errors that could arise as a result of aDNA damage.

#### Sex determination

To determine the genetic sex of the ancient individuals, we calculated the coverage on the autosomes as well as on each sex chromosome and subsequently normalized the X and Y reads by the autosomal coverage^107^. For that, we used a custom script (https://github.com/TCLamnidis/Sex.DetERRmine) for the calculation of each relative coverage as well as their associated error bars^108^. Females are expected to have an X rate of 1 and a Y rate of 0, whereas males are expected to have a rate of 0.5 for both X and Y chromosomes.

#### Contamination estimation

We used the ANGSD (analysis of next generation sequencing data) package^109^ (v.0.923) to test for heterozygosity of polymorphic sites on the X chromosome in male individuals, applying a contamination threshold of 5% at the results of method 1. For male and female samples, we estimated contamination levels on the mtDNA using Schmutzi^110^ (v.1.5.4) by comparing the consensus mitogenome of the ancient sample to a panel of 197 worldwide mitogenomes as a potential contamination source, applying a contamination threshold of 5%.

#### Genotyping

We used the program pileupCaller (v.1.4.0.2) (https://github.com/stschiff/sequenceTools.git) to genotype the trimmed BAM files of ten UDG half libraries. A pileup file was generated using samtools mpileup with parameters -q 30 -Q 30 -B containing only sites overlapping with our capture panel. From this file, for each individual and each SNP on the 1,240k panel^39,40,111^, one read covering the SNP was drawn at random and a pseudohaploid call was made; that is, the ancient individual was assumed homozygous for the allele on the randomly drawn read for the SNP in question. For the 15 single-stranded libraries that underwent no UDG treatment, we used the parameter -SingleStrandMode, which causes pileupCaller to ignore reads aligning to the forward strand at C/T polymorphisms and at G/A polymorphisms to ignore reads aligning to the reverse strand, which should remove postmortem damage in aDNA libraries prepared with the non-UDG single-stranded protocol. To maximize our resolution, we filled missing data in the single-stranded libraries with additional genotypes present in the trimmed, double-stranded libraries but not in the single-stranded libraries.

#### Mitochondrial and Y chromosome haplogroup assignment

To process the mtDNA data, we extracted reads from 1,240k data using samtools (v.1.3.1)^112^ and mapped these to the revised Cambridge reference sequence. We subsequently called consensus sequences using Geneious R9.8.1 (ref. ^113^) and used HaploGrep 2 (v.2.4.0)^114^ (https://haplogrep.uibk.ac.at/; with PhyloTree v.17-FU1) to determine mitochondrial haplotypes. For the male individuals, we used pileup from the Rsamtools package to call the Y chromosome SNPs of the 1,240k SNP panel (mapping quality ≥30 and base quality ≥30). We then manually assigned Y chromosome haplogroups using pileups of Y-SNPs included in the 1,240k panel that overlap with SNPs included on the ISOGG SNP index v.15.73 (Y-DNA Haplogroup Tree 2019-2020; 2020.07.11).

#### Identity-by-descent

We imputed and phased individuals with >500,000 SNPs (OAK003B, OAK007, OAK012 and OAK013) using GLIMPSE^115^ (v.2.0.0) (https://github.com/odelaneau/GLIMPSE), applying the default parameters and using the 1,000 genomes reference panel. Samples with >600,000 SNPs exhibiting a genotype posterior of ⩾0.99 after imputation were included in downstream IBD analysis. Subsequently, we used BEAGLE^116,117^ (v.5.2) to phase the newly imputed genotypes. Following ref. ^118^, the window and overlap lengths were set as wider than any chromosome (window length 380 cM and overlap length 190 cM) to maximize the information used for phasing the genomes. The 1,000 genomes phase 3 dataset (https://bochet.gcc.biostat.washington.edu/beagle/1000_Genomes_phase3_v5a) and GRCh37 genomic maps (https://bochet.gcc.biostat.washington.edu/beagle/genetic_maps/) provided by BEAGLE were used for phasing. The identification of IBD segments was done using RefinedIBD^119^. The window size was set to 3 cM. The minimal size for a segment to be considered shared by IBD is 1 cM, the same threshold used in refs. ^118,120^. Finally, we removed gaps between IBD segments that have at most one discordant homozygote and that are <0.6 cM in length and aggregated the sum and number of IBD segments between each pair of ancient and present-day individuals.

#### Kinship estimation

We calculated the pairwise mismatch rate^121^ in all pairs of individuals from our pseudohaploid dataset to double-check for potential duplicate individuals and to determine first-, second- and third-degree relatives. However, no relatives were identified.

#### Diversity estimation

CND was estimated by counting the differences between the ascertained pseudohaploid genotype calls present in one pair of individuals from the same population as described in the section on ‘Kinship estimation’ above. For these comparisons we grouped the individuals per geographic origin and time period. Results are reported as boxplots, where each dot corresponds to the CND value for a unique pair of individuals. We also estimate average heterozygosity levels for the imputed genomes of OAK007, OAK012 and OAK013 as well as for two published Iron Age individuals from Botswana (XAR001 and XAR002) by taking the fraction of the number of heterozygous sites over the total number of sites across 22 autosomes^122^. Subsequently, we compared these estimates with the heterozygosity levels observed in 562 individuals belonging to 49 present-day sub-Saharan African populations.

### Population genetic analysis

#### Dataset

We merged our aDNA data with previously published datasets of ancient individuals reported by the Reich Lab in the Allen Ancient DNA Resource v.54.1 (https://reich.hms.harvard.edu/allen-ancient-dna-resource-aadr-downloadable-genotypes-present-day-and-ancient-dna-data) (1,240k SNP panel)^123^ (Supplementary Table 26). Present-day data from primarily sub-Saharan Africans were assembled from refs. ^7,8,36,111,124^ (human origins SNP panel and human origins-Schlebusch SNP panel) (Supplementary Table 27). We excluded recently admixed individuals for PCA, DYSTRUCT and qpAdm analysis from ref. ^7^ (see Data Availability).

#### Naming

Within tables and figures, we refer to populations by the names given in the Allen Ancient DNA resource v.54.1 (ref. ^123^). Additionally, we refer to individuals from Oakhurst, St. Helena^2^, Faraoskop^2^ and Ballito Bay^1^ grouped together as South Africa LSA or SA_LSA. We follow the San Council recommendations in using population-specific terms whenever possible and alternatively use the terms San for Tuu and K’xaa language-speaking hunter-gatherer groups and Khoe for Khoe-Kwadi speakers. Within the text, we spell names of San and Khoe groups using click consonant symbols. Within figures and tables we refer to populations using the labels from the original publications of the genotype data. When necessary we collectively refer to groups with indigenous southern Africa-specific ancestry as having San-related ancestry. We used the label ‘coloured’ for some groups in the figures and supplementary tables following the labelling in the original publications of these genomes^1,7^. This bureaucratic denotation refers to South Africans of mixed ancestry, who represent a biologically heterogeneous group with variable and complex admixture from indigenous San and Khoe, European, Bantu-speaking African, Asian and Madagascan Cape slaves or migrants^60^. In the text, we use the label South Africans of mixed ancestry for these individuals, acknowledging that many are genetically homogenous to one origin yet were classified during apartheid under a single racial label, which is still used today^60^.

#### Principal components analysis

We carried out PCA using the smartpca software v.16000 from the EIGENSOFT package (v.6.0.1)^125^. We computed principal components on two different sets of southern African populations and projected ancient individuals using lsqproject: YES and shrinkmode: YES. Dataset (1) includes 24 San, Khoe and Bantu-speaking populations from three sources (refs. ^7,8,111^) as well as 212,000 SNPs (Fig. 1b); dataset (2) includes only 22 San, Khoe and Bantu-speakining populations from two sources (refs. ^25,111^) but 597,000 SNPs (Extended Data Fig. 1b). We highlight that the PCA computed on dataset (1) better reflects the genetic diversity within southern San (which is under-represented in dataset (2) because of the lack of samples from South Africa).

#### *F*-statistics

*F*_3_- and *F*_4_-statistics were computed with ADMIXTOOLS v.3.0 (ref. ^35^) (https://github.com/DReichLab). *F*_3_-statistics were calculated using qp3Pop (v.435). For *F*_4_-statistics, we used the qpDstat (v.755) and with the activated *F*_4_-mode. Significant deviation from zero can be interpreted as rejection of the tree population typology ((Outgroup, X);(Pop1, Pop2)). Under the assumption that no gene flow occurred between Pop1 and Pop2 and the Outgroup, a positive *F*-statistic suggests affinity between X and Pop2, whilst a negative value indicates affinity between X and Pop1. Standard errors were calculated with the default block jackknife 5 cM in size. As outgroup for *F*_3_- and *F*_4_-statistics, we used either diploid genotypes from two archaic human genomes (a Neanderthal^126^ and a Denisovan^127^) or haploid genotypes from one chimpanzee genome (the chimpanzee genome is required for technical reasons as an outgroup to all humans).

#### Fixation index

We calculated *F*_ST_ using smartpca software v.16000 from the EIGENSOFT package (v.6.0.1)^125^ with the fstonly, inbreed and fsthiprecision options set to YES.

#### Inference of mixture proportions

We estimated ancestry proportions using qpWave^39,128^ (v.410) and qpAdm^39^ (v.810) from ADMIXTOOLS v.3.0 (ref. ^35^) with the allsnps: YES and inbreeding: YES options and one basic set of 11 outgroups modified from ref. ^33^: Mbuti, Dinka, Ju_hoan_North, Turkey_N^129^, Iran_GanjDareh_N^40^, French, Sardinian, Punjabi, Ami, Papuan and Karitiana.

For group-based qpAdm analysis, we tested for each ancient and present-day population 1-, 2- and 3-way admixtures scenarios between SA_LSA (consisting of Oakhurst, without OAK006, St. Helena, Faraoskop and Ballito Bay), Tanzania_Luxmanda_3000BP and Mende.DG^2^. We selected for each population the admixture model with *P* > 0.01 featuring the lowest number of sources.

To analyse potential sex bias in the admixture process, we used qpAdm to estimate SA_LSA admixture proportions on the autosomes (default option) and on the X chromosome (option “chrom: 23”) using the abovementioned outgroups. Following the approach established by ref. ^42^, *Z*-scores were calculated for the difference between the autosomes and the X chromosome using the formula *Z* = $\frac{{{\mathrm{pA}}}-{{\mathrm{pX}}}}{\sqrt{{\mathrm{\sigma {A}}}^{2}+{{\mathrm{\sigma X}}}^{2}}}$ where pA and pX are the SA_LSA admixture proportions on the autosomes and the X chromosome and *σ*A and σX are the corresponding jackknife standard deviations^42^. Thus, a negative *Z*-score means that there is more SA_LSA admixture on the X chromosome than on the autosomes, indicating that the SA_LSA admixture was female-biased.

#### Ancestry decomposition

We performed model-based clustering analysis using two different approaches: (1) We applied DYSTRUCT^30^, with a cluster number (*K*) ranging between 2 and 10. Mean radiocarbon ages for the ancient individuals included were converted to generation ages (assuming a generation time of 30 yr; refs. ^130,131^) and provided for the analysis. (2)We applied ADMIXTURE^63^ in supervised mode using modern reference populations at *K* = 7. This analysis was run on haploid data with the parameter –haploid set to all (="*"). To obtain point estimates for populations, we averaged individual point estimates and calculated the s.e.m. As modern references we used the following groupings: San (Ju_hoan_North.DG, Khomani_San.DG), West Africa (YRI.SG, ESN.SG), East Africa (Somali, Masai, Sandawe), South Europe (TSI.SG, IBS.SG), North Europe (CEU.SG, GBR.SG), South Asia (PJL.SG, GIH.SG), East Asia (CHB.SG, JPT.SG)^111,132,133^. The *Q* matrix of this ADMIXTURE analysis was also used as input for FSTruct as described by the authors^64^.

#### Potential ascertainment bias

Recent analyses have shown that co-modelling more than one sub-Saharan African and/or archaic human groups (Neanderthals and Denisovans) using *F*-statistics in a non-outgroup-ascertained SNP panel can lead to false rejection of true demographic histories and failure to reject incorrect models in *F*_4_-derived methods like qpAdm^134^. However, most *F*_4_-statistics themselves remain minimally affected by ascertainment^134^. Regarding qpAdm analysis, we compared our results calculated on the complete 1,240k or human origins SNP panels with the published results by ref. ^2^. That study^2^ obtained an outgroup-ascertained set of 814,242 transversion SNPs polymorphic between the Denisovan and Neanderthal genomes to minimize the bias on *F*-statistics^2^. We find our qpAdm estimates to be highly correlated with the ones reported by Skoglund et al.^2^ (Extended Data Fig. 7a) (Pearson’s product-moment correlation; *t* = 22.244, d.f. = 17, *P* = 5.23 × 10^−14^, cor = 0.983), suggesting a negligible effect of ascertainment bias on our results. The qpWave analysis was restricted to A/T and G/C sites in the 1,240k SNP panels as recommended by ref. ^134^.

#### Admixture dating

Admixture dates between SA_LSA and Tanzania_Luxmanda, Mende or English as sources were calculated using DATES (distribution of ancestry tracts of evolutionary signals) (v.4010)^88^ using standard settings. A default bin size of 0.001 M is applied in our estimates (flag “binsize: 0.001” added). We used a standard of 29 years per generation to convert the generation times in years since admixture.

#### Maximum likelihood tree

We constructed maximum likelihood trees using TreeMix (v.1.12)^25^. For each tree, we performed a round of global rearrangements after adding all populations (-global) and calculated 100 bootstrap replicates to assess the uncertainty of the fitted model (-bootstrap). Sample size correction was disabled.

### Reporting summary

Further information on research design is available in the Nature Portfolio Reporting Summary linked to this article.

## Supplementary information

Reporting Summary

Supplementary Tables Supplementary Tables 1–27.

## Data Availability

Raw sequence data (fastq files) and mapped data (bam files) from the 13 newly reported ancient individuals will be available before publication from the European Nucleotide Archive under accession no. PRJEB77188. A poseidon package of the genotype data analysed in this paper is available on the Poseidon Community Archive (https://www.poseidon-adna.org/#/archive_explorer). Owing to ethical prescriptions of this research under UCT human research ethics no. 715/2017, DNA sequencing libraries, both before and after SNP capture, are available for replication upon request to the corresponding authors and the UCT Human Skeletal Repository Committee at uctskeletalrepository-group@uct.ac.za as aliquots, pending consultation, approval and permission by the UCT Skeletal Repository Committee and consulted San communities who granted the original sample access. Previously published genotype data for ancient and present-day individuals were reported by the Reich Lab in the Allen Ancient DNA Resource v.54.1 (https://reich.hms.harvard.edu/allen-ancient-dna-resource-aadr-downloadable-genotypes-present-day-and-ancient-dna-data). Additional previously published genotype data for the present-day San and Khoe samples from ref. ^7^ are available at the Arrayexpress database (https://www.ebi.ac.uk/arrayexpress/) under accession no. E-MTAB-1259. The Genome Reference Consortium Human Build 37 (GRCh37/hg19) is available via the National Center for Biotechnology Information under accession no. PRJNA31257. The revised Cambridge reference sequence is available via the National Center for Biotechnology Information under reference sequence NC_012920.1.

## Code availability

All software used in this work is publicly available. List of software and respective versions: AdapterRemoval (v.2.3.1), Burrows–Wheeler Aligner (v.0.7.12), DeDup (v.0.12.2), mapDamage (v.2.0.6), BamUtil (v.1.0.14), EAGER (v.1), Sex.DetERRmine (v.1.1.2) (https://github.com/TCLamnidis/Sex.DetERRmine), ANGSD (v.0.915), Schmutzi (v.1.5.4), PMDtools (v.0.50), pileupCaller (v.1.4.0.2), samtools (v.1.3.1), Geneious (R9.8.1), HaploGrep 2 (v.2.4.0), READ (https://bitbucket.org/tguenther/read) (v.f541d55), PLINK (v.1.90b3.29), Picard tools (v.2.27.3), smartpca (v.16000; EIGENSOFT v.6.0.1), qp3Pop (v.435; ADMIXTOOLS v.3.0), qpDstat (v.755; ADMIXTOOLS v.3.0), qpWave (v.410), qpAdm (v.810), DATES (v.4010), ADMIXTURE (v.1.3), GLIMPSE (https://github.com/odelaneau/GLIMPSE) (v.2.0.0), DyStruct (https://github.com/tyjo/dystruct) (v.2.0.0), BEAGLE (v.5.4), RefinedIBD (v.17Jan20.102), FSTruct (https://github.com/MaikeMorrison/FSTruct) (d39827e) and TreeMix (v.1.12). Data visualization and descriptive statistical tests were performed in R (v.4.1.1). The following R packages were used: Rsamtools (v.2.12.0), vegan (v.2.6-2), factoextra (v.1.0.7), ggplot2 (v.3.3.6), ggExtra (v.0.10.0), ggforce (v.0.3.3), rnaturalearth (v.0.1.0), sf (v.1.0.-8), raster (v.3.5-21), rgdal (v.1.5-32), spatstat (v.2.3-4), maptools (v.1.1-4), gstat (v.2.0-9), sp (v.1.5-0), labdsv (v.2.0-1), rcarbon (v.1.5.1), magrittr (v.2.0.3), dplyr (v.1.0.9), reshape 2 (v.1.4.4) and tidyverse (v.1.3.2). Y chromosome and mtDNA haplogroups were determined using the ISOGG SNP index (v.15.73) and PhyloTree (v.17-FU1) reference databases, respectively.
